# Intraoperative femoral fracture during uncemented total hip arthroplasty: does the stem length matter?

**DOI:** 10.1177/11207000251404013

**Published:** 2026-01-08

**Authors:** Ibrahim Almazrua, Dimitris Dimitriou, Peter Staunton, David Zukor, Olga Huk, John Antoniou

**Affiliations:** Division of Orthopaedic Surgery, SMBD-Jewish General Hospital and McGill University, Montreal, Quebec, Canada

**Keywords:** Microplasty, periprosthetic fracture, taperloc stem, total hip arthroplasty

## Abstract

**Background:**

Short stems have been developed to conserve bone stock, especially in the younger population undergoing total hip arthroplasty (THA). They demonstrated functional outcomes comparable to conventional stems. The purpose of this study was to compare the incidence of acute periprosthetic femoral fractures (PPFx) during posterior and lateral THA, between stems of the same manufacturer (Taperloc Microplasty (stem A) versus Taperloc complete (stem B)).

**Methods:**

Institution’s database was searched for all primary THA performed between August 2016 and August 2023. Preoperative x-rays were analysed to characterize the proximal femoral geometry, specifically the canal bone ratio (CBR) and canal flare index (CFI). Data analysis was performed to identify risk factors for PPFx.

**Results:**

2107 femoral stems (stem A: 1727, stem B: 380) were implanted. 53% were women. The average age was 70 ± 11 years. PPFx rate was 0.94%, with 20 PPF (stem A: 17, stem B: 3). There was no significant difference in PPFx rates between the 2 stems (0.98% vs. 0.79%, *p* > 0.72) The multivariate regression analysis demonstrated that stem length, CBR, CFI, age and gender were not risk factors for PPFx.

**Conclusions:**

Taperloc Microplasty and complete stems had similar rates of PPFx, and stem length was not a risk factor for a PPFx during uncemented THA.

## Introduction

Uncemented femoral stems in total hip arthroplasty (THA) have been demonstrating excellent long-term outcomes.^1–3^ Nevertheless, current literature and research continue to strive to improve on mechanical properties, designs, and biological characteristics of current THA prostheses. As a result, short stem concept designs have gained popularity through many unique advantages, such as bone preservation, which is a very important factor when revision arthroplasty is required for any reason.^4^ Also, they optimise proximal load transfer, producing better bone integration and less bone resorption.^5^ Moreover, due to the short design of the implant, there was a theoretical advantage of easier insertion through smaller incisions and fewer complications, including fractures, compared to regular-length stems ^6^.

Recent literature suggests that stem length might be an independent risk factor for acute periprosthetic femoral fracture (PPFx) in direct anterior THA or with different short stem designs.^7,8^. However, different short stems can potentially have different geometry, therefore stems with similar features and different lengths are required to exclude potential confounding factors and investigate the role of implant length in acute periprosthetic fracture.

The Taperloc system (Zimmer Biomet, Warsaw, IN, USA) includes the regular length Complete and the Microplasty stem, which is 35 mm shorter. Both stems have the same proximal flat tapered wedge geometry, which in turn can enhance proximal offloading and provide rotational stability. It is made of titanium alloy with 2 offset options including standard and high offset, with a 133° neck-shaft angle, as well as a coxa vara option. Both stems have a tapered design with a plasma spray porous coating to increase the surface area of the implant and allow bony ingrowth. The purpose of the present study was to compare the incidence of acute periprosthetic femoral fractures, between Microplasty and Complete, THA performed through the posterior and lateral approach.

## Methods

### Study design, inclusion, and exclusion criteria

The present study was approved by the Hospital ethical committee and was entirely conducted at the authors’ institution. Consecutive patients who underwent a primary THA from August 2016 to August 2023 were identified. Inclusion criteria were adult patients, who underwent a cementless THA with the Taperloc Microplasty or Taperloc complete femoral stem for symptomatic osteoarthritis (primary or secondary) and had completed at least a 1-year follow-up (including a pelvic anteroposterior and cross-table lateral radiograph) following THA at the time of the data collection. Exclusion criteria were revision hip arthroplasty, of any kind.

### Patient characteristics

Stem selection and surgical approach were based on institutional preference at the time of surgery or individual surgeon preference. All patients undergoing uncemented THA by any of the 3 author surgeons were included in the study. Institutional preference was the Taperloc system. There were no criteria for selection of short versus standard stem, but both were available. Selection was based on surgeon preference with 2 of 3 surgeons utilising the short stem almost exclusively. The third surgeon used the short stem exclusively for an initial period and then converted to the standard stem based on concerns regarding PPFx. Surgeons used individual methods, based on perceived bone mineral density and age, to determine the choice of uncemented versus cemented implants on a case-by-case basis.

Pre-selected data including patient demographics, clinical profile, and surgical details were extracted and anonymised. Periprosthetic fractures occurring intraoperatively or within 1-year post-op were identified using our arthroplasty database. Preoperative x-rays were reviewed, and femoral morphology was assessed by measuring the canal bone ratio (CBR), and canal flare index (CFI). Preoperative templating was not performed.

### Implants studied

The implants investigated in the present study were the Taperloc Microplasty and Taperloc Complete (Zimmer Biomet, Warsaw, IN, USA). The 2 femoral stems are tapered, highly porous, proximally coated, cementless, and collarless and have identical proximal femoral characteristics with the only difference being the length of the stem (and the broach) which is 35 mm longer in the Taperloc complete. The length of both stems increases with increasing stem size. The Microplasty stem length ranges from 93 mm up to 132 mm, and the Tapecloc complete from 128 mm to 154 mm.

### Data analysis

Descriptive data for categorical variables were summarised with frequencies while continuous parameters were described by averages, ranges, and standard deviations. Comparisons among categorical data (gender, side, type of osteoarthritis, surgical approach, and rate of periprosthetic fractures) were evaluated using Pearson’s chi-square test. All continuous parameters (age, follow-up, CBR, and CFI) were tested with the Kolmogorov-Smirnov test for normality. A 2-tailed unpaired *t*-test was then used to compare this data between groups (stem A vs. stem B). A univariate regression analysis was performed to identify whether patient demographics (age, gender), diagnosis, surgical approach, proximal femoral morphology (CBR, CFI), or stem length were associated with periprosthetic fractures. All the statistical analyses were performed using SPSS version 23 software (SPSS Inc., Chicago, IL, USA).

## Results

A total of 2107 THA (Men: 990, Women: 1117) with an average age of 70 ±11 years were identified (Table 1). A total of 1727 stem A and 380 stem B were implanted during the selected time period. The average follow-up in patients with stem A and stem B was 53 ± 22 (range 12–89) months and 20 ± 6 (range 12–77) months, respectively. 7% were lost to follow-up, including deceased patients. A total of 22 (1%) periprosthetic fractures occurred during the assessed time period. The periprosthetic fracture rate was similar between groups at 1% (Table 2). In the Microplasty group a total of 18 fractures were observed, 12 intraoperative and 6 within the first 6 weeks postoperative (Table 3). In the Complete group, a total of 4 fractures has been observed, 3 intraoperative and 1 at 2 weeks postoperative. Fracture rates were comparable among the 3 surgeons, with no statistical significance. We did not find any overlap in stem length between the largest short and the smallest long stems. The largest short stem we used was size 20 (length 125 mm) and the shortest long was size 5 (length 130 mm).

**Table 1.** Patient demographics. The values are given in average value and range.

Patient demographics	All (*n* = 2107)	Stem A (*n* = 1727)	Stem B (*n* = 380)	Significance (*p*-value)
Age, average ± SD (range), years	70 ± 11 (26, 103)	71 ± 11 (26, 103)	69 ± 11 (34, 93)	*n.s*
Male gender, *n* (%)	994 (47)	805 (47)	189 (50)	*n.s*
Right side, *n* (%)	1122 (53)	907 (53)	215 (57)	*n.s*
Osteoarthritis				
• Primary, *n* (%)	2016 (96)	1643 (95)	373 (98)	*n.s*
• AVN, *n* (%)	46 (2)	43 (2.4)	3 (0.7)	*n.s*
• DDH, *n* (%)	20 (1)	20 (1)	0	*n.s*
• Inflammatory, *n* (%)	25 (1)	21 (1)	4 (1)	*n.s*
Approach				
• Direct lateral, *n* (%)	1015 (48)	1010 (58)	5 (1)	<0.001*
• Posterior, *n* (%)	1091 (52)	716 (42)	375 (99)	<0.001*
Follow-up, average ± SD (range), months	47 ± 24 (12, 89)	53 ± 22 (12, 89)	20 ± 6 (12, 77)	<0.001*

*n.s*, not significant; AVN, avascular necrosis; DDH, developmental dysplasia of the hip; SD, standard deviation.

Note: Values in bold **i**ndicate a statistically significant difference between the Taperloc Microplasty (stem A) and the complete (stem B) *(*p* < 0.05).

**Table 2.** Fracture group characteristics. The values are given in average value and range.

Fracture group characteristics	Stem A (*n* = 18)	Stem B (*n* = 4)	Significance (*p*-value)
Periprosthetic fracture, *n* (%)	18 (1)	4 (1)	*n.s*
Age, average ± SD (range), years	70 ± 10 (54, 91)	76 ± 16 (55, 89)	*n.s*
Male gender, *n* (%)	6 (33)	0	*n.s*
Right side, *n* (%)	8 (44)	1 (25)	*n.s*
Osteoarthritis			
• Primary, *n* (%)	16 (90)	4 (100)	*n.s*
• AVN, *n* (%)	1 (5)	0	*n.s*
• DDH, *n* (%)	0	0	*n.s*
• Inflammatory, *n* (%)	1 (5)	0	*n.s*
Approach			
• Direct lateral, *n* (%)	7 (39)	0	<0.001*
• Posterior, *n* (%)	11 (61)	4 (100)	<0.001*
CBR	0.41 ± 0.1 (0.2, 0.7)	0.42 ± 0.1 (0.3, 0.5)	*n.s*
CFI	4.2 ± 0.8 (2.9, 6.6)	4.2 ± 0.4 (4, 4,7)	*n.s*

*n.s*, not significant; SD, standard deviation; AVN, avascular necrosis of the femoral neck; CBR, canal bone ratio; CFI, canal flare index; DDH, developmental dysplasia of the hip.

Note: Values in bold **i**ndicate a statistical difference *(*p* *<* 0.05).

**Table 3.** Fracture types.

	Classification	Femoral implant	Timing	Management
1	A2 (intra)	Stem A	Intraoperative	Tension band wiring
2	B3	Stem A	6 weeks post-op	Stem exchange
3	A2 (intra)	Stem A	Intraoperative	Cables
4	B2	Stem A	Intraoperative	Cables
5	A2 (intra)	Stem A	Intraoperative	Cables
6	B2	Stem A	Intraoperative	Cables
7	A2 (intra)	Stem A	Intraoperative	Cables
8	B3	Stem A	Day 1 postoperative	Stem exchange
9	C2	Stem A	Day 5 postoperative	Stem exchange
10	B2	Stem A	Intraoperative	Cables
11	B2	Stem A	Intraoperative	Cables
12	B2	Stem A	Intraoperative	Cables
13	B3	Stem A	6 weeks post-op	Stem exchange
14	B2	Stem A	Intraoperative	Cables
15	B3	Stem A	4 weeks post-op	Stem exchange
16	B2	Stem A	Intraoperative	Cables
17	B2	Stem A	Day 4 postoperative	Stem exchange
18	B2	Stem A	Intraoperative	Cables
19	B3	Stem B	2 weeks post-op	Stem exchange
20	B2	Stem B	Intraoperative	Cables
21	B2	Stem B	Intraoperative	Cables
22	A2	Stem B	Intraoperative	Cables

The univariate regression analysis showed no correlation between periprosthetic fracture and patient demographics (age, gender), diagnosis, approach, proximal femoral morphology (CBR, CFI), or stem length.

## Discussion

The debate continues regarding the superiority of short stems compared to standard stems in terms of complications and the possible advantages such as preserving bone stock, better bone integration, and less bone resorption. The purpose of the present study was to investigate the relationship between stem length and periprosthetic fracture rate among stems with similar geometry and broaching systems but with different broach/stem lengths. The major finding of the present study was that both stems demonstrated similar rates of periprosthetic fractures, and the stem length was not a risk factor for a periprosthetic fracture during uncemented THA.

The relationship between the femoral stem length and periprosthetic fracture rate has been scarcely investigated in the literature with contradictory results. Barrington et al.^9^ compared the clinical and radiographic outcomes in 849 Taperloc Complete and 902 Taperloc Microplasty stems and reported a fracture rate of 0.3% and 0.6% (*p* > 0.05), respectively. However, they found that early periprosthetic fractures were more likely to occur in elderly patients. On the other hand, Molli et al.^6^ found a statistically significant fracture rate in standard-length stems compared to short ones, among 658 THA from which 269 had short stems. A recent study performed by our group,^8^ comparing 1561 Taperloc Microplasty and 1631 Tri-lock bone preserving stems, reported a 4 times higher fracture rate in the Taperloc Microplasty (0.96% versus 0.25%) and that stem length as an independent risk factor for periprosthetic fracture.

**Figure 1.**
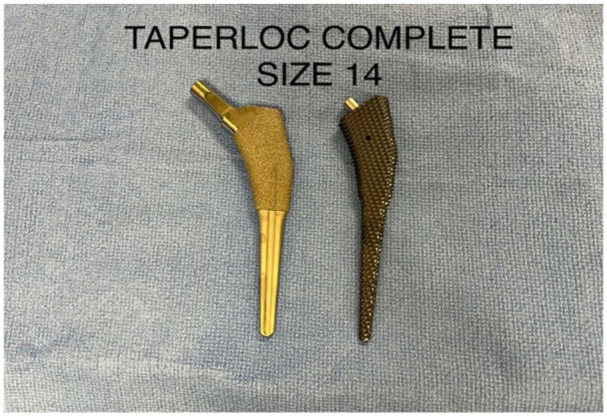
Taperloc Complete.

**Figure 2.**
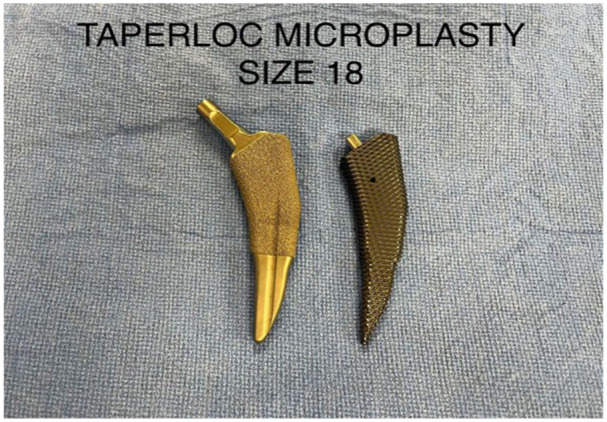
Taperloc Microplasty.

Intraoperative fractures typically occur during either broaching or implant insertion. Broach design differs from implant philosophy and can be classified as compaction, blunt extraction, and sharp extraction type. Smooth tamps have been shown to pose a higher risk of intraoperative fracture when compared to toothed broaches.^10^ Broach tooth or step geometry dictates the coefficient of friction, and radial forces transmitted to the bone during broaching are theoretically higher with compaction or blunt extraction type broaches compared to a sharp extraction type broach.^11^ Within our cohort, most of the periprosthetic fractures occurred intraoperatively or within the first week following the operation without significant trauma, suggesting occult intraoperative fracture (75% in Complete and 83% in Microplasty, *p* > 0.05). Since both stems use sharp extraction broaches with similar geometry (except for the length) the similar rate of periprosthetic fractures between the 2 stems was not surprising (Figures 1 and 2). This finding is contrast to the Tri-lock bone preserving stem system that uses a blunt extraction broach which we reported to have a significantly lower (0.25%) periprosthetic fracture rate.^8^ A difference that could be due to the different broach type or to the slightly different stem geometry. Moreover, it is important to note that increasing the stem size, regardless of whether it is short or long stem, results in an increase not only in length but also in width and diameter. The medial curvature remains constant while the lateral dimensions grow with each size. A noteworthy point that warrants further investigations.

Patient demographics and surgical approach have been investigated as potential risk factors for periprosthetic fracture. Our results showed no correlation between age and fracture rate. However, others have found links between older age and fracture rate.^9,12^ Compared to Barrington et al.^9^ paper, the average age and ranges were similar in both of our studies, but there is likely some heterogeneity in patient selection. Our institutions approach to the use of cemented components in at risk patients may have reduced the risk of fracture in older patients when compared to the Barrington group. We also had more fractures in the Microplasty group but this was not statistically significant for our group. The shorter stem used non-selectively predisposes to a higher likelihood of proximal femoral morphology-implant mismatch, and therefore a higher fracture-rate in those at highest risk of mismatch. Tanzer et al.^13^ reviewed the Australian Orthopaedic Association National Joint Replacement Registry data. Comparing the best 3 cemented with the best 3 uncemented stems in patients ⩾75 years. The authors concluded that the overall early complication rate, including fractures, were higher in cementless group compared to cemented group.^13^ Regarding surgical approach, some studies found increased risk of periprosthetic fractures in direct anterior approach (DAA) whereas others reported no differences. A multicentre retrospective review by Meneghini et al.^14^ demonstrated a higher percentage of periprosthetic fractures in the anterior approach compared to either the posterior or lateral approach, which was reaffirmed by another single-centre study.^15^ On the other hand, Lygrisse et al.^16^ reported similar rates of periprosthetic fractures in DAA (0.7%), posterior (0.63%) and lateral approach (0.68%, *p* > 0.05), which has also been supported by other studies ^8,17^. In our study, the surgical approach (posterior vs lateral) was not a risk factor for a periprosthetic fracture. Similar to other studies,^9,18^ the preoperative diagnosis did not have any impact on the perioperative fracture rate.

The present study should be interpreted in light of its potential limitations, mostly inherent to the retrospective design. However, due to the standardized clinical and radiological follow-up protocol most of the patient data we needed was available for the current analysis. However, an accurate assessment of intraoperative fracture timing (i.e., during broaching or final implant insertion) was not achievable due to the absence of accurate documentation of the individual events. Finally, the present study investigated 2 types of stems with similar proximal geometry and broaching systems but with different lengths to minimise potential confounders in evaluating the relationship between stem length and periprosthetic fracture rates. As such, the current results may not be extrapolated to all stem designs. However, we do belief that the study is helpful in terms of advancing the discussion regarding implant factors that contribute to an increased risk of PPF, given our attempt to control for one variable, length.

In conclusion, stem length was not a risk factor for PPF during uncemented THA in the stems examined. By isolating stem length within a single design, this study minimised confounding variables; however, the findings may not be generalised to other stem types.

## References

[1] Ellison B Berend KR Lombardi AV Jr , et al. Tapered titanium porous plasma-sprayed femoral component in patients aged 40 years and younger. J Arthroplasty 2006; 21(Suppl. 2): 32–37. 16950059 10.1016/j.arth.2006.03.008

[bibr2-11207000251404013] Emerson RH Jr Head WC Emerson CB , et al. A comparison of cemented and cementless titanium femoral components used for primary total hip arthroplasty: a radiographic and survivorship study. J Arthroplasty 2002; 17: 584–591. 12168174 10.1054/arth.2002.32696

[3] Lombardi AV Jr Berend KR Mallory TH , et al. Survivorship of 2000 tapered titanium porous plasma-sprayed femoral components. Clin Orthop Relat Res 2009; 467: 146–154. 18975042 10.1007/s11999-008-0568-x PMC2600990

[4] Santori FS Santori N. Mid-term results of a custom-made short proximal loading femoral component. J Bone Joint Surg Br 2010; 92: 1231–1237. 20798440 10.1302/0301-620X.92B9.24605

[5] Chen HH Morrey BF An KN , et al. Bone remodeling characteristics of a short-stemmed total hip replacement. J Arthroplasty 2009; 24: 945–950. 18848420 10.1016/j.arth.2008.07.014

[6] Molli RG Lombardi AV Jr Berend KR , et al. A short tapered stem reduces intraoperative complications in primary total hip arthroplasty. Clin Orthop Relat Res 2012; 470: 450–461. 21971877 10.1007/s11999-011-2068-7 PMC3254753

[7] Tamaki T Jonishi K Miura Y , et al. Cementless tapered-wedge stem length affects the risk of periprosthetic femoral fractures in direct anterior total hip arthroplasty. J Arthroplasty 2018; 33: 805–809. 29107490 10.1016/j.arth.2017.09.065

[8] Staunton P Alhojailan K Desgagne C , et al. Acute periprosthetic hip fractures with short, uncemented femoral stems. J Arthroplasty 2024; 39(S1): S248–S253. 10.1016/j.arth.2024.05.087 38851408

[9] Barrington JW Emerson RH Jr. The short and “shorter” of it: >1750 tapered titanium stems at 6- to 88-month follow-up. J Arthroplasty 2013; 28(Suppl.): 38–40. 24034508 10.1016/j.arth.2013.07.032

[10] Kold S Mouzin O Bourgeault C , et al. Femoral fracture risk in hip arthroplasty: smooth versus toothed instruments. Clin Orthop Relat Res 2003; 408: 180–188. 10.1097/00003086-200303000-00023 12616057

[11] Batz J Syrigos S Vorbeck M , et al. The influence of broach design on bone friction and osseodensification in total hip arthroplasty. Clin Biomech (Bristol, Avon) 2020; 73: 234–240. 10.1016/j.clinbiomech.2019.12.012 32062473

[12] Greco NJ Lombardi AV Jr Morris MJ , et al. Direct anterior approach and perioperative fracture with a single-taper wedge femoral component. J Arthroplasty 2019; 34: 145–150. 30301574 10.1016/j.arth.2018.09.003

[13] Tanzer M Graves SE Peng A , et al. Is cemented or cementless femoral stem fixation more durable in patients older than 75 years of age? A comparison of the best-performing stems. Clin Orthop Relat Res 2018; 476: 1428–1437. 29683803 10.1097/01.blo.0000533621.57561.a4 PMC6437589

[14] Meneghini RM Elston AS Chen AF , et al. Direct anterior approach: risk factor for early femoral failure of cementless total hip arthroplasty: a multicenter study. J Bone Joint Surg Am 2017; 99: 99–105. 28099299 10.2106/JBJS.16.00060

[15] Aggarwal VK Elbuluk A Dundon J , et al. Surgical approach significantly affects the complication rates associated with total hip arthroplasty. Bone Joint J 2019; 101-B: 646–651. 31154834 10.1302/0301-620X.101B6.BJJ-2018-1474.R1

[16] Lygrisse KA Gaukhman GD Teo G , et al. Is surgical approach for primary total hip arthroplasty associated with timing, incidence, and characteristics of periprosthetic femur fractures? J Arthroplasty 2021; 36: 3305–3311. 34016522 10.1016/j.arth.2021.04.026

[17] Sershon RA McDonald JF 3rd Ho H , et al. Periprosthetic femur fracture risk: influenced by stem choice, not surgical approach. J Arthroplasty 2021; 36(7S): S363–S366. 10.1016/j.arth.2021.02.012 33736894

[18] McLaughlin JR Lee KR. Total hip arthroplasty with an uncemented tapered femoral component in patients younger than 50 years of age: a minimum 20-year follow-up study. J Arthroplasty 2016; 31: 1275–1278. 26781396 10.1016/j.arth.2015.12.026

